# Novel splice-site variants in *TMPRSS3* impair hearing via exon skipping and abrogated protease activity

**DOI:** 10.3389/fgene.2026.1791840

**Published:** 2026-03-18

**Authors:** Xiang Wang, Wei-Qian Wang, Sha-Sha Huang, Xi Wang, Li-Min Hu, Xing Liu, Qiu-Quan Wang, Zhen-Dong Wang, Jin-Cao Xu, Yong-Yi Yuan, Xue Gao

**Affiliations:** 1 Postgraduate Training Base of Jinzhou Medical University: The PLA Rocket Force Characteristic Medical Center, Beijing, China; 2 Department of Otolaryngology, PLA Rocket Force Characteristic Medical Center, Beijing, China; 3 Senior Department of Otolaryngology Head and Neck Surgery, the 6th Medical Center of Chinese PLA General Hospital, Chinese PLA Medical School, Beijing, China; 4 State Key Laboratory of Hearing and Balance Science, Beijing, China; 5 National Clinical Research Center for Otolaryngologic Diseases, Beijing, China; 6 Key Laboratory of Hearing Science, Ministry of Education, Beijing, China; 7 Beijing Key Laboratory of Hearing Impairment Prevention and Treatment, Beijing, China

**Keywords:** ARNSHL, DFNB8/10, hearing loss, splice-site variant, TMPRSS3

## Abstract

**Background:**

Hearing loss (HL) is genetically heterozygous, making its genetic diagnosis challenging. Identification of novel HL-associated genes and variants will enhance our understanding of the molecular mechanisms and improve genetic diagnosis. *TMPRSS3*, encoding a transmembrane serine protease, is implicated in autosomal recessive nonsyndromic hearing loss (ARNSHL), designated as DFNB8 (postlingual onset) or DFNB10 (prelingual onset). Although over 100 pathogenic *TMPRSS3* variants have been reported, only seven splice-site variants have been documented to date.

**Aims/objectives:**

This study investigates the molecular etiology of ARNSHL in three Chinese family and functionally characterizes five novel *TMPRSS3* variants, including one non-canonical splice-site variant, two canonical splice-site variants, and two missense variants.

**Material and methods:**

Whole-exome sequencing and gene panel sequencing identified candidate variants, followed by validation with Sanger sequencing. Functional analyses included minigene splicing assays to evaluate the variants’ effect on mRNA splicing and a yeast-based functional assay to assess their impacts on protease activity.

**Results:**

Three families carrying compound heterozygous *TMPRSS3* variants were identified. The proband with c.205 + 5G>C/c.923T>C (p.Met308Thr) presented with progressive, postlingual, high-frequency–predominant, nonsyndromic sensorineural hearing loss, consistent with DFNB8. Probands with c.572 + 1G>A/c.967G>A (p.Val323Met) and c.1348-2A>G/c.271C>T (p.Arg91Ter) exhibited prelingual, nonsyndromic sensorineural hearing loss. Functional studies revealed that all five novel variants (c.205 + 5G>C, c.923T>C, c.572 + 1G>A, c.967G>A, and c.1348-2A>G) led to reduced protease activity. Notably, the splice-site variants caused exon skipping and resulted in a more pronounced loss of activity compared to the missense variants.

**Conclusion and significance:**

This study expands the spectrum of *TMPRSS3* variants and provides mechanistic insights into their pathogenicity. Intriguingly, splice-site variants led to a more severe impairment of enzymatic activity compared to missense variants, providing molecular evidence for the considerable genotype-phenotype heterogeneity observed in *TMPRSS3*-associated hearing loss.

## 1 Introduction

Hearing loss (HL) is among the most prevalent sensory disorders worldwide, with genetic factors contributing to over 50%–80% of congenital HL cases (Shearer et al., 1993; Choe et al., 2023). The extreme genetic heterogeneity of HL, involving hundreds of genes, poses significant challenges for molecular diagnosis and genetic counseling. Autosomal recessive nonsyndromic hearing loss (ARNSHL) accounts for approximately 70%–80% of hereditary HL cases, with 88 associated genes identified to date (Hereditary Hearing Loss Homepage, accessed, 10/19/25).

*TMPRSS3* (MIM *605511), which encodes a transmembrane serine protease, is an important causative gene associated with both prelingual (DFNB10, MIM #605316) and postlingual (DFNB8, MIM #601072) forms of ARNSHL. *TMPRSS3*-related hearing loss is highly heterogeneous in severity, ranging from mild to profound, and typically follows a progressive course. Pathogenic variants in this gene are among the top ten genetic cause of HL (Sang et al., 2019). The TMPRSS3 protein is expressed in key cochlear cell types, including hair cells, supporting cells, the stria vascularis, and the spiral ganglion neurons (Chen et al., 2022; Guipponi et al., 2002; Arora et al., 2025; Tang et al., 2019).

To date, over 100 pathogenic *TMPRSS3* variants have been reported, including missense, nonsense, frameshift, and splice-site alterations (Nisenbaum et al., 2023). Among these, splice-site variants remain exceptionally rare, with only seven documented cases (Gao et al., 2017a; Lechowicz et al., 2017; Imtiaz et al., 2011; Richard et al., 2019). The phenotypic severity of HL is influenced by both the type and genomic location of the variant, with splice-site and missense variants often exerting distinct functional effects (Guipponi et al., 2008).

Despite advances in genetic testing, molecular diagnosis of HL remains challenging due to the large number of candidate genes and the presence of variants of uncertain significance (VUS). Functional validations are therefore essential, particularly for variants suspected to affect splicing or enzymatic activity. Although a few *TMPRSS3* splice-site variants have been reported, their precise molecular mechanisms and genotype-phenotype correlations are not fully understood (Boudewyns et al., 2018; Kim et al., 2017).

In this study, we investigated three Chinese families with ARNSHL and identified five novel compound heterozygous *TMPRSS3* variants. The variants include one non-canonical splice-site variant (c.205 + 5G>C), two canonical splice-site variants (c.572 + 1G>A, c.1348-2A>G), and two missense variants (c.923T>C, p. Met308Thr, c.967G>A, p. Val323Met). We performed functional analyses—minigene splicing assays and yeast-based protease activity assays—to assess their pathogenicity. Our findings expand the mutational spectrum of *TMPRSS3* and provide new mechanistic insights into the genotype-phenotype correlations underlying DFNB8/10-related HL.

## 2 Materials and methods

### 2.1 Study subjects

This retrospective study utilized in-house databases of genetic hearing loss from the College of Otolaryngology Head and Neck Surgery at the Chinese PLA General Hospital. The clinical assessment for HL included medical history collection (via questionnaire), otoscopy, pure tone audiometry, acoustic immittance, and stapedius reflexes. HL severity was classified according to the 2021 WHO guidelines, based on the average pure tone audiometry (PTA) threshold at 0.5, 1, 2, and 4 kHz (mild: 26–40 dB HL; moderate: 41–60 dB HL; severe: 61–80 dB HL; profound: ≥81 dB HL). Frequency-specific HL was defined as low (125–500 Hz), mid (1–2 kHz), and high (4–8 kHz). Prelingual (onset before age 3) and postlingual (onset after age 3) HL were distinguished. Vestibular function was evaluated using tandem gait, Romberg, and caloric test, while temporal bone CT were also assessed.

### 2.2 Whole exome sequencing (WES) and variant analysis

Whole-exome sequencing (WES) and targeted gene panel sequencing were performed for each proband. Sequence reads were aligned to the GRCh38 reference genome, followed by variant calling and annotation. Variant filtering was prioritized on known HL-related genes. Candidate variants were classified based on American College of Medical Genetics and Genomics (ACMG)/Association for Molecular Pathology (AMP) guidelines for genetic hearing loss and validated by Sanger sequencing (Richards et al., 2015).

### 2.3 Construction of *TMPRSS3* minigene plasmids

Three *TMPRSS3* splice-site variants (c.205 + 5G>C, c.572 + 1G>A, and c.1348-2A>G) were functionally tested using the pSPL3 exon-trapping vector (Miaolinbio, Wuhan, China) (Westin et al., 2021). Genomic DNA from HEK293T cells served as the template for generating mutant fragments by PCR using gene-specific primers (Supplementary Table S1, TsingkeBio, Beijing, China). For the c.205 + 5G>C mutant, exon 3 along with 338 bp of the upstream intron and 476 bp of the downstream intron were amplified. The first PCR (primers 205-F1 and 205-R1) amplified exon 3 with a 338 bp segment of intron 2, while the second PCR (primers 205-F2 and 205-R2) amplified exon 3 with a 476 bp segment of intron 3. These two fragments were then fused by overlap-extension PCR using primers 205-F1 and 205-R2 to generate the full-length mutant construct. The same strategy was applied for constructing the c.572 + 1G>A mutant fragment (exon 5 with 335 bp of upstream intron and 442 bp of downstream intron) and the c.1348-2A>G mutant fragment (exon 13 with 450 bp of upstream intron and 396 bp of downstream intron).

For cloning, the pSPL3 plasmid was digested with EcoRI and NotI (NEB, US). The ligation ratio was determined using the online tool provided by Vazyme (https://crm.vazyme.com/cetool/restructure.html). The ligation products were transformed into competent *E. coli* DH5α cells (Takara, Japan) and plated onto ampicillin-containing agar plates, followed by overnight incubation at 37 °C. Single colonies were picked and inoculated into LB medium supplemented with ampicillin, then cultured for 3 h. Colony PCR and Sanger sequencing were performed using primers SD-6 and SA2 to confirm correct insertions. Colonies with correct constructs were expanded in 8 mL of ampicillin-containing LB medium overnight at 37 °C with shaking. Plasmid DNA was subsequently extracted using the EndoFree Plasmid Midi Kit (CWbio, Beijing, China).

### 2.4 Cell transfection and splicing assay

HEK293T cells were cultured in DMEM (Thermo, US) supplemented with 10% fetal bovine serum (FBS) and penicillin-streptomycin (Beyotime, Shanghai, China) and seeded into 12-well plates. After 24 h, the cells were transfected in triplicate with either wild-type or mutant pSPL3 plasmids (c.205 + 5G>C, c.572 + 1G>A, and c.1348-2A>G) using the jetPRIME® transfection system (Polyplus, Shanghai, China), with 0.8 μg DNA, 1.6 μL jetPRIME® reagent, and 75 μL jetPRIME® buffer per well. Total RNA was extracted 48 h post-transfection using a kit (ABclonal, Beijing, China) and reverse-transcribed into cDNA (ABclonal, Beijing, China).

PCR amplification was performed in a 40 μL reaction volume. The cycling conditions were as follows: 95 °C for 12 min; 35 cycles of denaturation at 95 °C for 30 s, annealing at 60 °C for 30 s, and extension at 72 °C for 30 s; followed by a final extension at 72 °C for 5 min. The PCR products were separated on a 2% agarose gel at 160 V for 15 min and visualized under UV light. The remaining products were subjected to Sanger sequencing to confirm the splicing alterations resulting from the variants.

### 2.5 Molecular cloning and construction of wild-type (WT) and mutant *TMPRSS3* expression plasmids

After confirming that splice-site variants alter mRNA expression, wild-type *TMPRSS3* cDNA was obtained from HeLa cell (which express TMPRSS3 protein according to the Human Protein Atlas, www.proteinatlas.org). Mutant *TMPRSS3* constructs were subsequently generated by overlap extension PCR using gene-specific primers (see Supplementary Table S1). For functional assays in yeast, wild-type and mutant *TMPRSS3* sequences were cloned into the pGBKT7 vector (carrying a TRP1 auxotrophic marker), and the TMPRSS3 substrate epithelial sodium channels (ENaC, Coolaber, Beijing, China) was cloned into the pGADT7 vector (carrying a LEU2 auxotrophic marker). Following transformation and selection, yeast strains of each genotype were plated onto YPD medium, incubated, and colony numbers were quantified using the ImageJ software. The experiments were performed in triplicate, and the collected data are presented as mean ± SD. Statistical significance was analyzed using one-way ANOVA followed by Tukey’s *post hoc* test.

## 3 Results

### 3.1 Clinical findings

**FIGURE 1:**
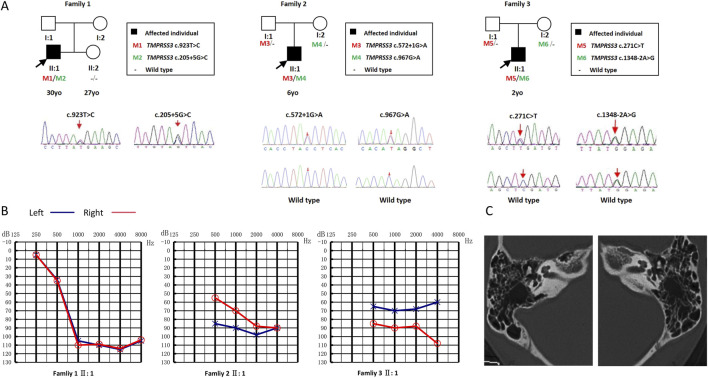
Pedigree, audiograms, temporal bone CT of families with *TMPRSS3* causatives. **(A)** Family 1: WES identified compound heterozygous *TMPRSS3* variants c.205 + 5G>C and c.923T>C (p.Met308Thr); the spouse tested negative for common deafness genes. Family 2: A hearing-loss gene panel detected c.572 + 1G>A and c.967G>A (p.Val323Met). Family 3: WES revealed c.1348-2A>G and c.271C>T. **(B)** Audiological findings. Family 1 II:1: Hearing loss noted at age 10; audiogram obtained at 24 years. Family 2 II:1: First audiological assessment at 1 year 2 months. Family 3 II:1: Hearing impairment observed after age 1; preoperative thresholds recorded at 2 years 10 months before right cochlear implantation. **(C)** Temporal-bone CT of the Family 1 proband. CT demonstrated patent external auditory canals, normal tympanic membranes, well-aerated middle ears, and symmetrical internal auditory canals without stenosis or dilation.

Family 1: the proband (II:1, a 30-year-old Han Chinese male, Figure 1A) presented with progressive, postlingual, severe sensorineural HL with onset at age 10 (Figure 1B). Audiograms revealed mid-to-high-frequency HL with absent stapedius reflexes. Tinnitus was reported. Vestibular function tests (tandem gait, Romberg, caloric tests) were unremarkable. There were no structural abnormalities of the outer, middle or inner ear were observed on temporal bone CT (Figure 1C). The patient currently uses bilateral hearing aids for auditory rehabilitation. No family history of HL was reported.

Family 2: the proband (II:1, a 6-year-old Han Chinese male) presented with prelingual, severe sensorineural HL. The patient underwent left cochlear implantation at 1 year and 2 months of age, and the audiogram represents his preoperative hearing status (Figure 1B). Following implantation, his auditory and speech-language development demonstrated significant improvement. No family history of HL was reported.

Family 3: the proband (II:1, a 10-year-old Han Chinese male) presented with progressive, prelingual, severe sensorineural HL with onset at age 1. The patient underwent right cochlear implantation at 2 years and 10 months of age (Figure 1B). The presented audiogram, obtained preoperatively, documents the baseline hearing level prior to surgery. His postoperative listening and language abilities have shown marked improvement. No family history of HL was reported.

### 3.2 Identification of *TMPRSS3* variants

**FIGURE 2:**
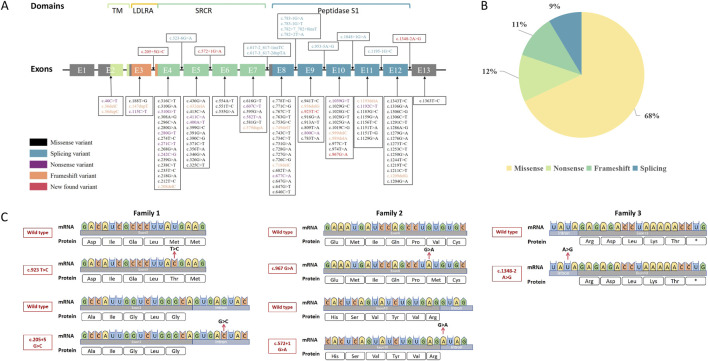
Summary of reported and novel *TMPRSS3* variants. **(A)** Distribution of known and novel *TMPRSS3* variants. Previously reported variants were compiled from HGMD and PubMed and mapped across the gene (see Supplementary Table S2). **(B)** Proportion of variant types. Incorporating five novel variants from this study, the overall distribution was: missense 68%, nonsense 12%, frameshift 11%, and splice-site 9%. **(C)** Novel variants identified in this study. Comparative mRNA and protein sequence diagrams illustrate the local molecular changes for each family.

Previously reported *TMPRSS3* variants were compiled from the Human Gene Mutation Database (HGMD) and cross-referenced with the PubMed literature. These data were then integrated to construct a comprehensive map of known *TMPRSS3* variants (Figure 2A). The retrieved variants, detailed in Supplementary Table S2, revealed that splice-site variants are considerably less frequent than missense variants. Among all documented variants, missense variants constituted the majority (68%), followed by nonsense (12%), frameshift (11%), and splice-site (9%) variants (Figure 2B). In this study, we identified five novel variants, comprising three splice-site variants and two missense variants (Figure 2C). WES identified three novel *TMPRSS3* variants c.205 + 5G>C, c.923T>C (p.Phe308Leu), and c.1348-2A>G with robust sequencing quality (median depth >100× for c.205 + 5G>C and c.923T>C, >200× for c.1348-2A>G; >98–99% of targeted regions covered at ≥30×). Based on ACMG/AMP guidelines, c.205 + 5G>C was classified as a variant of uncertain significance (PM2, PP3, PP4), c.923T>C as likely pathogenic (PM2, PM3, PP3, PP4), and c.1348-2A>G as pathogenic (PVS1, PM2, PM3, PP3, PP4, PP5). Panel sequencing revealed two additional novel variants with adequate coverage: c.572 + 1G>A (splicing), classified as pathogenic (PVS1, PM2, PM3, PP4), and c.967G>A, classified as a variant of uncertain significance (PM2, PM3, PP4).

The two missense variants are located in close proximity to each other, the c.923T>C variant results in a substitution of methionine by threonine at position 308 of the protein, and the c.967G>A variant leads to a substitution of valine by methionine at position 323. These variants alter a highly conserved residue within the catalytic serine protease domain. Located proximal to the most common pathogenic variant, c.916G>A, these variants are likely to exert similar deleterious effects (Gao et al., 2017a; Weegerink et al., 2011).

### 3.3 Splicing analysis

*In vitro* splicing assays were performed using pSPL3 plasmids carrying the splice site variants c.205 + 5G>C, c.572 + 1G>A, and c.1348-2A>G. To analyze the effect of each variant on splicing, we constructed minigenes containing either the wild-type or mutant sequences, which included the exons flanking the splice sites and portions of the adjacent introns. These constructs were transfected into HEK293T cells to evaluate splicing outcomes.

**FIGURE 3:**
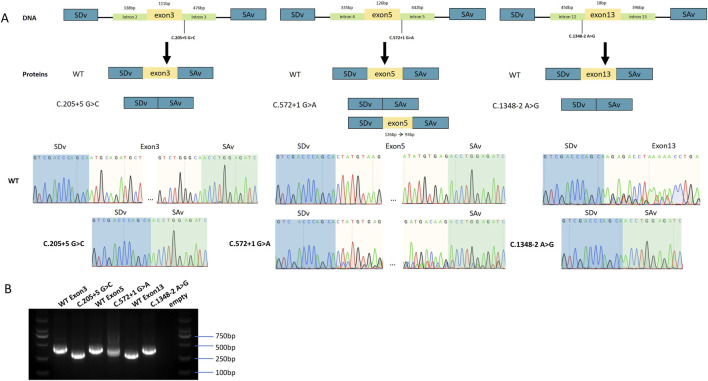
Analysis of the effects of splice-site variants using minigene assays. **(A)** Sequencing chromatograms of wild-type and mutant minigene constructs. SAv and SDv correspond to the upstream and downstream exons surrounding the inserted target gene. For c.205 + 5G>C, the minigene (338 bp upstream and 476 bp downstream of exon 3) showed normal splicing in the wild type and exon 3 skipping in the mutant. For c.572 + 1G>A, the construct (335 bp upstream and 442 bp downstream of exon 5) produced mixed transcripts in the mutant, including a 33 bp truncation and complete exon 5 skipping. For c.1348-2A>G, located downstream of the stop codon, the wild-type construct showed no SAv expression, whereas the mutant induced exon 13 skipping and restored SAv expression. **(B)** PCR results of wild-type and mutant minigenes. Gel electrophoresis showed paired bands for each exon: wild-type and mutant products for exon 3, exon 5, and exon 13, respectively.

Sequencing confirmed that the wild-type *TMPRSS3* fragments were correctly expressed, with proper splicing of exons 3, 5, and 13 (Figures 3A,B).

The c.205 + 5G>C is a non-canonical donor site variant in intron 2. It is predicted to cause skipping of exon 3, resulting in a 111 bp truncation of the transcript (Figure 3A). This event is predicted to disrupt the LDL receptor class A (LDLRA) domain, affecting protein stability.

The c.572 + 1G>A is a canonical donor site variant in intron 5 within the scavenger receptor cysteine-rich (SRCR) domain. This variant results in two splicing outcomes: complete skipping of the exon 5, similar to c.205 + 5G>C, resulting in a 126 bp truncation of the transcript; or a 33 bp truncation within exon 5 (Figure 3A). Both outcomes are likely to induce misfolding of the highly conserved SRCR domain, leading to loss of enzymatic activity or impaired substrate recognition, thereby inhibiting ENaC activation.

The c.1348-2A>G is a canonical acceptor site variant upstream of exon 13 within the serine protease domain. It led to exon 13 skipping (Figure 3A). Since exon 13 contains the stop codon, its exclusion results in a frameshift and incorporation of downstream intronic sequences, leading to the formation of an aberrant serine protease (SP) domain.

Minigene assays confirmed that the splice-site variants led to aberrant splicing. While the wild-type construct produced the expected normal transcript, the mutant constructs resulted in abnormal splicing patterns. These findings were consistently replicated in three independent experiments in HEK293T cells. Sequencing all bands confirmed break points and splicing events.

### 3.4 Mutant TMPRSS3 proteins showed aberrant protease activity

Previous studies have demonstrated that TMPRSS3 cleaves ENaC, and the cleavage efficiency can serve as an indicator of TMPRSS3 enzymatic activity (Guipponi et al., 2002). Cleavage efficiency correlates with colony growth on selective medium. Yeast successfully co-transformed with the TMPRSS3 and its substrate plasmids carried the LEU and TRP markers, respectively, and were selected on minimal media containing 2% glucose lacking leucine and tryptophan (Leu^−^/Trp^−^). Subsequently, the Leu^+^/Trp^+^ transformants were then replica-plated onto YPD medium supplemented with 2% sucrose and 0.5 mg/mL antimycin A. Quantification of colony number and size was performed using the ImageJ software, serve as a proxy for protease activity (Kim et al., 2017; Wong et al., 2020).

**FIGURE 4:**
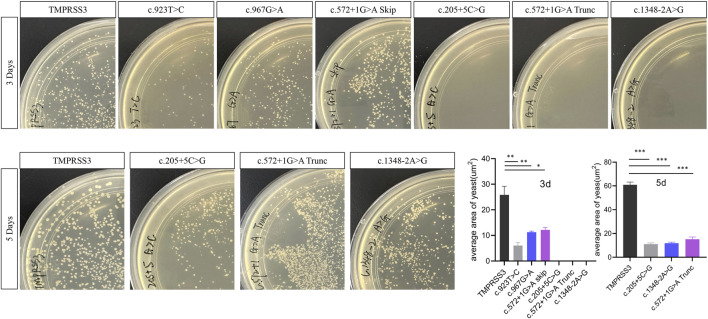
Functional assessment of *TMPRSS3* variants using a yeast protease assay. Colony growth was quantified using standard morphological criteria. After 3 days at 30 °C (top row), the assay shows the wild-type control followed by the missense variants c.923T>C and c.967G>A, and the splice-site variants c.572 + 1G>A, c.205 + 5G>C, and c.1348-2A>G. After 5 days (bottom row), the wild-type control and the three exon-skipping mutants (c.205 + 5G>C, c.572 + 1G>A, c.1348-2A>G) are shown. Colony numbers at both time points are presented in the accompanying graphs, demonstrating deleterious effects for all variants, with splice-site variants causing more severe growth impairment than missense variants. All experiments were performed with three independent biological replicates, and data are presented as mean ± SD. Statistical significance was analyzed using one-way ANOVA followed by Tukey’s *post hoc* test, with *p* < 0.05 considered statistically significant. *p < 0.05, **p < 0.01, ***p < 0.001.

To assess the pathogenic potential of the identified *TMPRSS3* variants, we evaluated the proteolytic activity of the mutant alleles in comparison to the wild-type protein. The results demonstrated a marked reduction in enzymatic activity across all mutant constructs. When colonies larger than 0.005 mm^2^ were quantified, those carrying the variants exhibited a significantly slower growth rate than the wild-type. By day 3, colonies harboring splice-site variants were scarcely visible, while those with missense variants were detectable but had a significantly smaller average area. Mutant colonies that remained undetectable by day 3 were further incubated alongside wild-type controls until day 5, at which point colonies corresponding to all three splice-site variants became visible, confirming their viability and excluding the possibility of complete growth failure. Collectively, these functional findings support the pathogenic potential of all five novel variants identified in this study (Figure 4).

While observing a decrease in protein activity, we also predicted the protein structural changes caused by each variant. Point variants did not cause obvious changes in the protein backbone, whereas splice-site alterations had a greater impact. However, predictions for the intramembrane and transmembrane regions differed markedly, and the confidence of the predictions was low (Supplement figure).

Interestingly, the colony growth defect associated with the non-canonical splice-site variant (c.205 + 5G>C) appeared less severe than that caused by the two canonical splice-site variants (c.572 + 1G>A and c.1348-2A>G) (Figure 4). This observation suggests a potential gradation in pathogenicity based on splice-site type, although further evidence is required to support this conclusion.

## 4 Discussion

In this study, we identified five novel compound heterozygous *TMPRSS3* variants (c.205 + 5G>C, c.923T>C, c.967G>A, c.572 + 1G>A, and c.1348-2A>G) in three Chinese individuals with prelingual autosomal recessive non-syndromic hearing loss (ARNSHL). The splice-site variants are particularly noteworthy, as they not only expand the mutational spectrum of *TMPRSS3* but also exhibit more severe pathogenic effects than the missense variants.

While over 100 *TMPRSS3* variants have been reported, splice-site variants remain exceptionally rare, with only seven had been clearly documented prior to this report. Our identification of three additional splice-site variants contributes significantly to this limited dataset. Functional characterization through minigene splicing assays confirmed that these variants disrupt normal mRNA processing, and protease activity assays demonstrated a marked reduction in enzymatic function. These findings extend beyond prior studies, which have predominantly focused on missense variants that impair protease activity, and provide further mechanistic support for establishing the genotype-phenotype correlations in *TMPRSS3*-related HL.

Yeast-based protease activity assays have served as an important functional tool for evaluating TMPRSS3 since 2003. Studies have consistently shown that pathogenic variants such as Asp103Gly, Arg109Trp, Cys194Phe, Trp251Cys, Pro404L, Cys407Arg, and Ala426Thr abolish yeast growth on sucrose plates, indicating a severe loss of proteolytic activity, whereas benign polymorphisms like Gly111Ser and Ile253Val do not impair function (Lee et al., 2003). Subsequent studies has corroborated this pattern, with variants like Val116Met and Val291Leu being highly deleterious, and others such as Arg80His, Leu184Ser, Ala418Val, and Trp251Cys also demonstrated severe functional impairment (Kim et al., 2017; Wong et al., 2020).

Previous work has shown that loss-of-function variants in any of the three major domains—LDLRA, SRCR domain, and serine protease—can result in HL (Guipponi et al., 2002; Masmoudi et al., 2001). In line with this, the variants identified in our study, including two missense and three splice-site alterations, all resulted in impaired protease function. Minigene assays specifically demonstrated that the splice-site variants c.205 + 5G>C, c.572 + 1G>A, and c.1348-2A>G caused the skipping of exons 3, 5, and 13, respectively. Notably, these splicing defects were associated with the most severe loss of enzymatic in our functional assays.

To date, 10 *TMPRSS3* splice-site variants have been associated with HL of varying severity. A pattern emerges where canonical splice-site variants (e.g., c.1195-1G>C and c.783-1G>A) generally cause prelingual, profound HL, likely due to complete exon skipping and protein truncation. In contrast, non-canonical variants such as c.323-6G>A and c.205 + 5G>C tend to produce postlingual or progressive phenotypes (DFNB8). This clinical distinction may be explained by partial splicing defects that preserve residual protein function, a hypothesis consistent with the possibility of incomplete exon skipping induced by non-canonical variants.

The pathogenic severity of *TMPRSS3* variants vary widely, and compound heterozygosity further complicates clinical interpretation. While missense variants have been more extensively studied, splice-site variants remain relatively under-investigated. Our study addressed this gap by functionally validation three novel splice-site variants, confirming they cause aberrant splicing and exon skipping, and demonstrating that all five novel variants impair proteolytic function. This provides direct molecular evidence of their pathogenicity.

The molecular pathogenesis underlying *TMPRSS3*-related HL have been elucidated through cellular and animal studies. Mice carrying a nonsense variant (*Tmprss3* Y206X) develop postnatal degeneration of cochlear hair cells beginning at postnatal day 12 (P12), demonstrating the protein’s essential role in hair cell survival and cochlear homeostasis (Shearer et al., 2025). Mechanistically, TMPRSS3 is known to activate the ENaC, maintains potassium homeostasis, and support normal outward K^+^ currents in inner hair cells. Taken together, these findings reinforce the critical functional role of *TMPRSS3* in the auditory system (Molina et al., 2013).

Globally, *TMPRSS3* variants account for approximately 0.36%–12% of genetic HL cases (Walsh et al., 2006; Wattenhofer et al., 2005; Damrongchietanon et al., 2025; Bharadwaj et al., 2025; Li et al., 2024), with notable differences among populations (e.g., only 0.36% in a large Japanese cohort) (Nisenbaum et al., 2023; Colbert et al., 2024; Moon et al., 2021; Gao et al., 2017b). Although not the most common ARNSHL gene, *TMPRSS3* remains clinically important due to: (i) its association with both prelingual (DFNB10) and postlingual (DFNB8) HL forms; (ii) phenotypic variability that correlates with variant type and domain; and (iii) its potential as a target for emerging gene therapies.

Recent advances in *TMPRSS3*-targeted gene therapy have been encouraging. Adeno-associated virus (AAV)-mediated gene replacement has successfully restored hearing in mouse models, paving the way for potential clinical translation (Du et al., 2023; Richard and Delprat, 2024). Our findings demonstrate that splice-site variants lead to adjacent exon skipping and that splice-site and missense variants differentially affect TMPRSS3 function, suggest that *TMPRSS3*-related HL may be amenable to tailored therapeutic strategies. Specifically, antisense oligonucleotide-based approaches designed to correct splicing defects could represent a viable treatment avenue for patients carrying splice-site variants.

## 5 Conclusion

In conclusion, our findings advance the understanding of *TMPRSS3*-related HL by functionally characterizing novel splice-site and missense variants and delineating their distinct impact on protein function. The more severe enzymatic impairment caused by splice-site variants provides a mechanistic explanation for the phenotypic heterogeneity observed in patients. This work underscores the necessity of precision medicine approaches in HL treatment, where therapeutic strategies may need to be tailored based on the specific molecular consequences of each patient’s variants. As gene therapies for HL advance toward clinical application, functional studies like this will be crucial for accurately matching patients with the appropriate therapeutic interventions. Integrating genetic diagnosis with detailed functional validation is a critical step toward achieving personalized medicine for hereditary hearing loss.

## Data Availability

The original contributions presented in the study are included in the article/supplementary material. The study’s WES data are not publicly available due China’s Regulation of Human Genetics Resource. Further inquiries can be directed to the corresponding authors.

## Supplementary material

SUPPLEMENTARY FIGURE S1 Functional assessment of *TMPRSS3* variants using a yeast protease assay. AlphaFold was used to perform 3D prediction and reconstruction of the protein structure. IPTM is the interface predicted template modeling score, PTM is the predicted template modeling score, and pLDDT is the predicted Local Distance Difference Test. (blue pLDDT > 90, cyan 90 > pLDDT > 70, yellow 70 > pLDDT > 50, and orange pLDDT < 50).
